# EPAC mediates the dual role of cAMP signaling in melanoma

**DOI:** 10.18632/oncoscience.463

**Published:** 2018-08-22

**Authors:** Carlos I. Rodriguez, Vijayasaradhi Setaluri

**Affiliations:** Department of Dermatology, University of Wisconsin-Madison, Madison, WI 53706, USA; William S. Middleton Memorial VA Hospital, Madison, WI 53705, USA

**Keywords:** cyclic AMP signaling, EPAC-RAP1 axis, BRAF/PTEN melanoma, melanoma progression

Cutaneous malignant melanoma arises from melanocytes, the pigment producing cells in the skin. Incidence of melanoma is increasing steadily during the past several decades. Melanoma is highly resistant to chemotherapy and patients with metastatic melanoma have a poor prognosis. Exposure to ultraviolet radiation (UV) from sunlight is the most common environmental risk factor for melanoma, although melanoma can occur in areas of the body not directly exposed to sun. Driver mutations in the mitogen-activated protein kinase (MAPK) pathway account for majority of melanomas [1]. Additionally, polymorphisms in the G-protein Coupled Receptor (GPCR) melanocortin-1 receptor gene *MC1R*, which are associated with fair skin and red hair, are risk factors for melanoma. These polymorphisms lead to defective repair of UV-induced DNA damage and reduced melanin production due to defective cyclic AMP (cAMP) signaling [2, 3].

Cyclic AMP signaling is also know to play a role in MAPK pathway activation and in the development of resistance to MAPK inhibition [4]. In melanoma, cAMP-dependent Protein Kinase A signaling was shown to cause cell cycle delay by inhibiting cdc25B and also activate the p53 tumor suppressor [5]. However, the role of cAMP in melanoma development and progression is not understood. Employing the Braf^CA^/Pten^−/−^ mouse melanoma model, we showed that topical application of the cAMP inducer, forskolin (FSK), accelerated melanoma tumor development [6]. Similarly, treatment of human primary melanoma cells *in vitro* with FSK stimulated their proliferation suggesting that cAMP signaling activation promotes melanoma tumor development and growth. Elevation of cAMP, however, inhibited the growth of metastatic melanoma cells indicating a dual role for cAMP signaling in melanoma.

The effects of activation of cAMP signaling on the proliferation of melanoma cells were not associated with the activation of Protein Kinase A and the cAMP Responsive Element Binding Protein (CREB), the classical effectors of cAMP signaling [6]. It is also worth noting that the effects of cAMP on growth of melanoma cells were independent of MAPK pathway activation status. Since cAMP is also known to signal through the alternative pathway involving Exchange Protein Directly Regulated by cAMP (EPAC), we investigated the possibility that EPAC proteins (EPAC1 and/or EPAC2) mediates the dual role of cAMP in melanoma. Consistent with the positive effects of cAMP elevation in primary melanoma, pharmacological inhibition of EPACs (with ESI-09, a cAMP competitive inhibitor of EPAC and EPAC2) markedly decreased the proliferation of human primary melanoma cells and stimulated the proliferation of metastatic melanoma cells. EPACs, also known as RAPGEFs, are guanine nucleotide exchange factors (GEF) for the Ras-related Proteins (Rap1/2). Small interference RNA-mediated inhibition of RAP1 mimicked the pro-and anti-proliferative effects of pharmacological inhibition of cAMP-EPAC, respectively, in primary and metastatic melanoma [6]. These data, therefore, showed that RAP1 is required for the effects of cAMP-EPAC signaling in melanoma. Although EPAC-RAP1 signaling is known to activate AKT via PI3K signaling [7], modulation of EPAC-RAP1 signaling in melanoma did not alter AKT activation status. Based on these data, we propose that during melanoma tumor progression, EPAC-RAP1 signaling axis acts to switch cAMP signaling from a pro-proliferative signal in primary melanoma to an anti-proliferative signal in metastatic melanoma.

**Figure 1:**
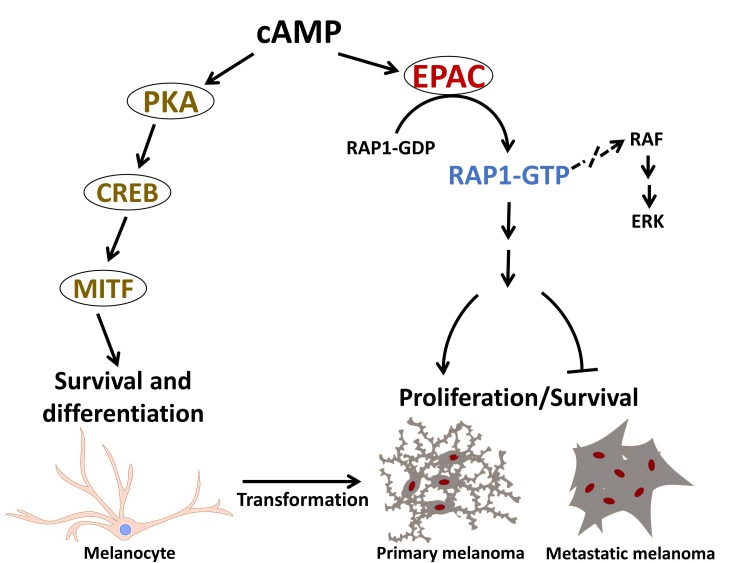
EPAC-RAP1 signaling axis promotes melanoma tumor development and mediates phenotype switching and the anti-proliferative response of metastatic melanoma to cAMP signaling Pharmacological inhibition EPAC and knockdown of RAP1 inhibit proliferation of primary melanoma cells and stimulate metastatic melanoma cells.

In the context of our data on anti-proliferative role of EPAC signaling in metastatic melanoma cells, it is interesting to note that EPAC has been reported to promote melanoma cell migration. These opposing roles of EPAC in proliferation and migration are consistent with the notion that proliferation is inhibited during tumor metastasis to favor an invasive phenotype. It has been documented that a similar phenotypic switch occurs in melanoma cell that converts them from a proliferative state to an invasive *in vivo* [8]. Taken together with the phenotype switching during melanoma progression, the opposite effects of EPAC signaling on proliferation of primary and metastatic melanoma cells suggest a critical role for EPAC signaling in phenotype switching in melanoma progression. We propose that EPAC and signaling proteins downstream of EPAC are attractive targets, respectively, for inhibition of melanoma progression and treatment of metastatic melanoma.
